# Safety of intracranial electrodes in an MRI environment: a technical report

**DOI:** 10.1002/jmrs.775

**Published:** 2024-03-11

**Authors:** Yarema B. Bezchlibnyk, Rolando Quiles, Jeremy Barber, Benjamin Osa, Keven Clifford, Ryan Murtaugh

**Affiliations:** ^1^ Department of Neurosurgery and Brain Repair, Morsani School of Medicine University of South Florida Tampa Florida USA; ^2^ Department of Radiology, Morsani School of Medicine University of South Florida Tampa Florida USA; ^3^ Department of Radiology Tampa General Hospital Tampa Florida USA; ^4^ PMT Corporation Chanhassen Minnesota USA; ^5^ Tower Radiology Tampa USA

**Keywords:** Displacement, intracranial leads, MRI, radiofrequency‐induced heating, safety, torque

## Abstract

**Introduction:**

Intracranial electroencephalography (iEEG) involves placing intracranial electrodes to localise seizures in patients with medically refractory epilepsy. While magnetic resonance imaging (MRI) enables visualisation of electrodes within patient‐specific anatomy, the safety of these electrodes must be confirmed prior to routine clinical utilisation. Therefore, the purpose of this study was to evaluate the safety of iEEG electrodes from a particular manufacturer in a 3.0‐Tesla (3.0T) MRI environment.

**Methods:**

Measurements of magnetically induced displacement force and torque were determined for each of the 10 test articles using standardised techniques. Test articles were subsequently evaluated for radiofrequency‐induced heating using a Perspex phantom in both open and ‘fault’ conditions. Additionally, we assessed radiofrequency (RF)‐induced heating with all test articles placed into the phantom simultaneously to simulate an implantation, again in both open and ‘fault’ conditions. Finally, each test article was evaluated for MRI artefacts.

**Results:**

The magnetically induced displacement force was found to be less than the force on the article due to gravity for all test articles. Similarly, the maximum magnetically induced torque was less than the worst‐case torque due to gravity for all test articles apart from the 8‐contact strip – for which it was 11% greater – and the depthalon cap. The maximum temperature change for any portion of any test article assessed individually was 1.7°C, or 1.2°C for any device component meant to be implanted intracranially. In the implantation configuration, the maximum recorded temperature change was 0.7°C.

**Conclusions:**

MRI may be safely performed for localising iEEG electrodes at 3.0T under certain conditions.

## Graphical abstract

This manuscript reports the results of an in vitro study to evaluate the safety and other issues associated with imaging intracranial electrodes, including stereo‐EEG depth electrodes and grid and strip electrodes, in a 3‐Tesla MRI environment. All measurements were performed according to American Society for Testing and Materials (ASTM) standards. The results of this study suggest that intracranial EEG electrodes may be imaged at 3T under certain conditions.

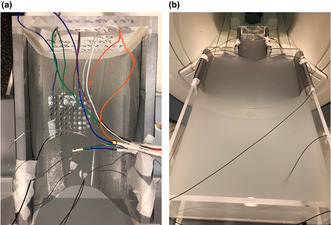

## Introduction

In intracranial electroencephalography (iEEG), multiple electrodes are placed intracranially to localise the seizure onset zone in patients with medically refractory epilepsy.^1^ Implantation strategies vary but may involve depth electrodes placed within the brain tissue (stereo electroencephalography (sEEG)) and subdural strip and/or grid electrodes placed on the brain surface. While magnetic resonance imaging (MRI) enables direct visualisation of electrodes relative to patient‐specific anatomy, important considerations include magnetically induced displacement and torque forces acting on the electrodes, radiofrequency (RF)‐induced heating of tissues in close proximity to the electrodes due to a local elevation in specific absorption rate (SAR), and artefacts arising from the electrodes which might limit the imaging study's spatial resolution.^2^ We quantified the impact of these issues on a variety of iEEG electrodes from a particular manufacturer to assess their safety in a 3.0‐Tesla (3.0T) MRI environment.

## Methods

### Intracranial electrodes

**Figure 1:**
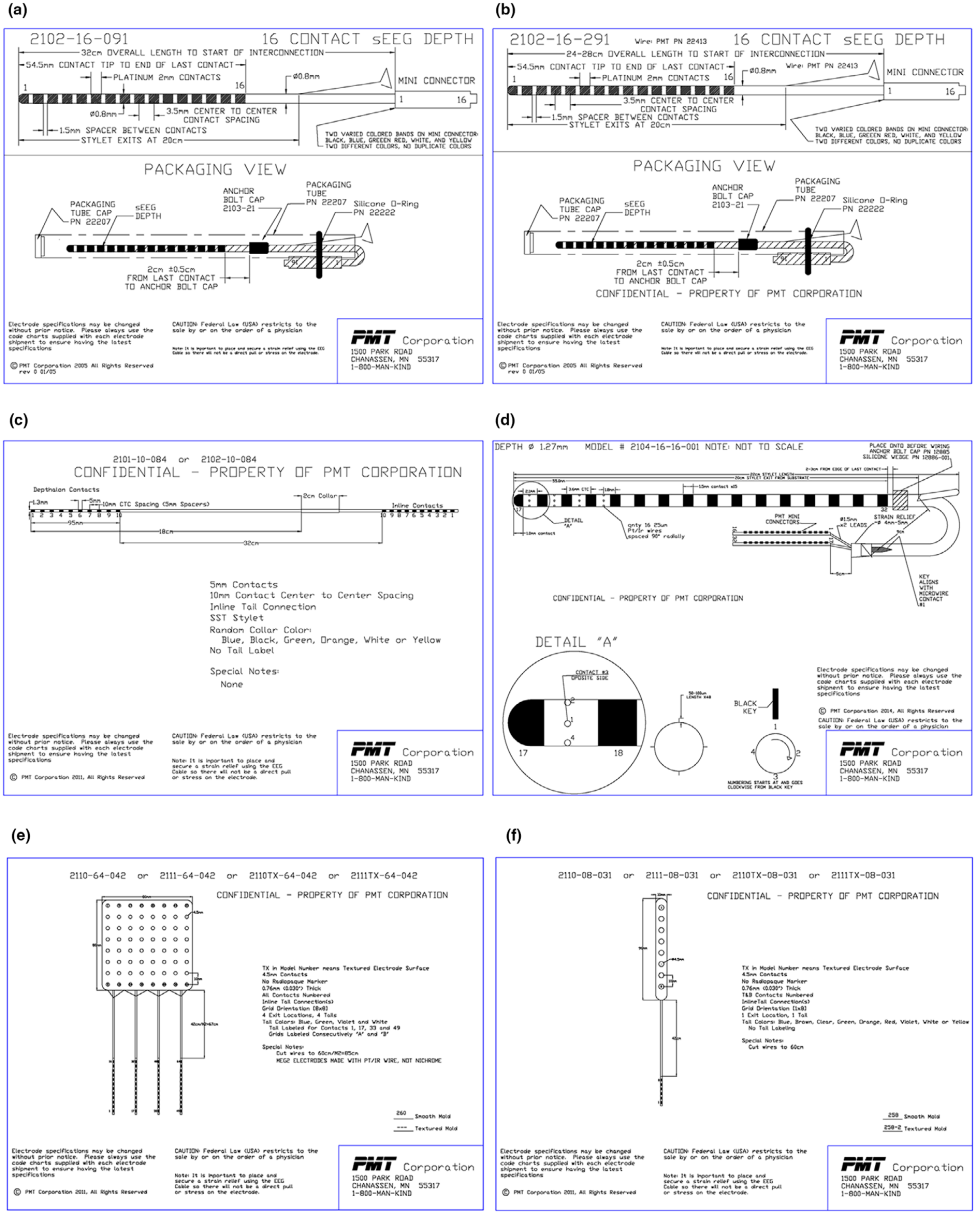
Specifications of test articles. (a) 16‐contact standard depth electrode ID# 2101‐16‐091, (b) 16‐contact RF ablation depth electrode ID# 2102‐16‐291, (c) 10‐contact depthalon depth electrode ID# 2102‐10‐084), (d) 8‐contact depth + 16‐contact microwire electrode ID# 2104‐16‐16‐001, (e) 64‐contact Contac grid ID# 2110‐64‐042, and (f) 8‐contact Contac strip ID# 2110‐08‐031. Permission was obtained from the copyright owner to reproduce these images.

A variety of depth and surface electrodes were provided by PMT Corporation (Chanhassen, MN, USA; Fig. 1, Figures [Link], [Link]).

### Phantom

**Figure 2:**
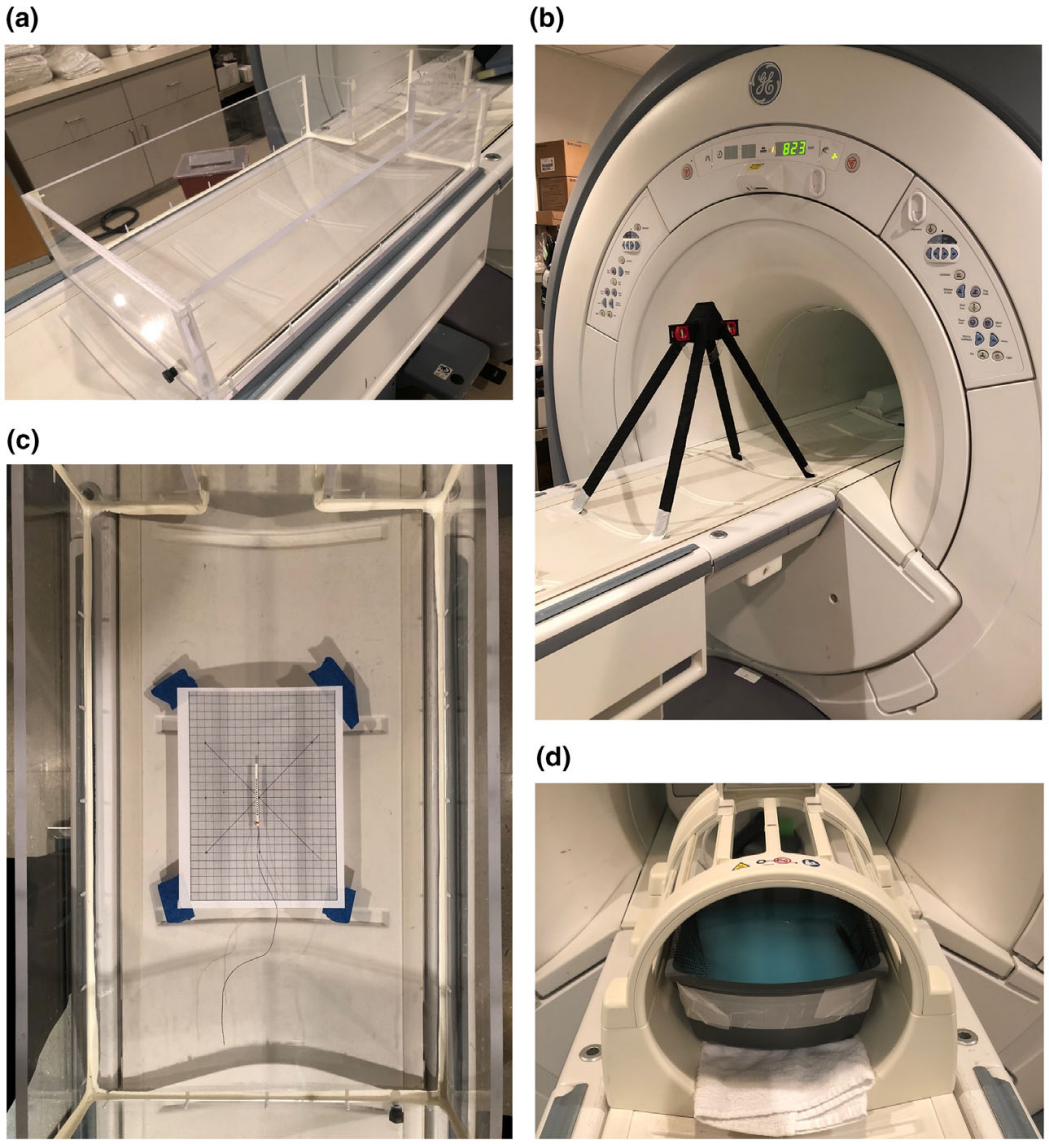
Pictures of experimental apparatuses. (a) Perspex phantom formed with a shape and dimensions approximating those of an adult human head and torso, (b) apparatus for measuring magnetically induced displacement, located at the point of greatest deflection of the test article (823 mm from isocenter), (c) picture of apparatus for measuring magnetically induced torque with the connector of the 16‐contact standard sEEG depth electrode (ID# 2101‐16‐091) positioned on the centre point of the grid and aligned along the *z*‐axis of the magnet, and (d) apparatus for evaluating MR image artefacts from passive implants. The device was suspended on a nylon netting within a plastic container and immersed in a 1.5 g/L solution of copper sulphate (CuSO_4_); an empty plastic pen was placed vertically into the solution as a reference.

A watertight Perspex phantom, similar to that described previously^3^, ^4^ and conforming to the American Society for Testing and Materials (ASTM) standard for testing passive implants in a magnetic resonance (MR) environment,^5^ was formed with a shape and dimensions approximating those of an adult human head and torso (Fig. 2a).

### MRI

Measurements were performed in a 3.0 T GE Signa HDxT MRI system (GE Healthcare, Chicago, IL, USA) using the transmit–receive body coil to approximate the worst‐case scenario, with all sources of air turned off. Fast spin echo (FSE) imaging was obtained in both anterior–posterior (AP) and right–left (RL) orientations for each test, according to the protocol: echo time (TE) = 14.0 ms, echoes = 1, repetition time (TR) = 425.0 ms, echo train length = 4, receiver bandwidth = 15.63 kHz; gradient mode = whole, imaging mode = 2D, pulse sequence = FSE‐XL, imaging options = fast; field of view (FOV) = 40.0 × 40.0 cm, slice thickness = 10.0 mm, slice spacing = 1.0 mm; slices = 40; matrix size = 256 × 256, and number of excitations (NEX) = 4.0. For RF‐induced heating, we performed 15‐min acquisitions and achieved an exposed body‐average SAR of 2.91 W/kg, based on an entered patient weight of 50 kg.

### Displacement

Each item was screened for ferromagnetic activity with a hand‐held ferrite magnet with a field strength of 108 Gauss at a height of 2 cm (magnet test). Next, the article was placed into a Perspex phantom resting on the MRI patient table, covered by a Perspex lid, and advanced towards isocenter (Phantom box test).

If no movement was observed on both screening tests, the item was evaluated for displacement in accordance with ASTM F2052‐15 – ‘Standard Test Method for Measurement of Magnetically Induced Displacement Force on Medical Devices in the Magnetic Resonance Environment’.^6^ Briefly, each item was suspended from a non‐ferromagnetic protractor by a polyester string affixed with paper tape first at the item's centre of mass, and next by its terminal ends. The assembly was placed onto the MRI patient table along the axis of the bore and brought towards the entrance (Fig. 2b). The magnetic field at the first movement was recorded. The item was then advanced to 823 mm from isocenter, where the spatial magnetic field gradient was between 80% and 100% of the maximum on the axis of the bore. Here, we recorded the angular deflection of the item from the vertical.

### Torque

If the item passed the displacement tests, it was evaluated for torque in accordance with ASTM F2213‐17 – ‘Standard Test Method for Measurement of Magnetically Induced Torque on Medical Devices in the Magnetic Resonance Environment’^7^ using the low‐friction surface method. Briefly, each item was placed on the centre point within the phantom and aligned along the *z*‐axis of the magnet; due to the irregular shape of these devices, we evaluated each test article when either the contacts or the connector segment were positioned on the centre point and aligned along the *z*‐axis of the magnet (Fig. 2c). The phantom was then moved to isocenter. The item was rotated 360^o^ in 45^o^ increments, and any realignment of the item was recorded at each increment. We repeated the test with the item placed on a piece of Tyvek® paper (Dupont, Wilmington, DE, USA) to minimise friction.

An upper bound on the magnetically induced torque on each item if no rotation was observed was obtained according to *T*_m_ < Lμmg (L = longest dimension, μ = coefficient of static friction, m = mass, and g = acceleration due to gravity), where μ = tanθ (θ = angle of repose).^7^ The angle of repose was obtained by placing the item on a Perspex surface, elevating it, and recording the angle at which the item is on the verge of sliding down. All measurements were obtained for the item as a whole and the contact and connector segments individually, and were performed three times for each item on bare Perspex and on Tyvek® paper.

### Radiofrequency‐induced heating

If the item passed the displacement and torque tests, it was evaluated for radiofrequency‐induced heating in accordance with ASTM F2182‐11 – ‘Standard Test Method for Measurement of Radio Frequency Induced Heating On or Near Passive Implants During Magnetic Resonance Imaging’,^5^ with modifications as indicated below. The phantom was filled to a depth of ~10 cm with a semi‐liquid gel comprising distilled water (99.499%), Carbomer 980 (0.498%), and magnesium nitrate (0.003%), with electrical characteristics similar to those of human tissue,^8^ including conductivity of 0.47 S/m and a specific heat capacity of 4160 J/(kg°C) at 21°C. The phantom was then placed within the MR suite and allowed to equilibrate with ambient temperature overnight. A lightweight nylon mesh was suspended within the gel, and the item(s) were placed onto the mesh, ensuring that no portion of any item was less than 2 cm from the gel surface or bottom of the container.

Initially, each item was assessed individually. Depth electrodes were oriented with the contacts along the left–right axis, while the subdural strip and grid electrodes were oriented with the contacts along the *z*‐axis to simulate the most common arrangement in clinical usage. In all cases, measurements were performed with two different tail configurations: (1) physically separated in an ‘open’ configuration; and (2) coiled, with the tail(s) forming a short circuit to simulate a ‘fault’ condition. The tail was then submerged within the gel and positioned parallel to the walls of the phantom. Continuous temperature measurements were made simultaneously from four positions using an MRI‐compatible fluoroptic thermometer (Model 3100; Luxtron Corporation, Santa Clara, CA, USA; accuracy ±0.1°C; SMM probes with 0.3 × 0.3 × 0.3 mm^3^ sensors). Probe #1 was placed at the most distal contact (contact #1) of the depth and subdural strip electrodes, and on the corner contact (contact #48) of the subdural grid electrode; the probe was oriented perpendicular to the long axis of the contact for depth electrodes or laid flat against the contact for strip or grid electrodes. Probe #2 was placed at the midpoint of the tail in the ‘open’ configuration, or where the tails contacted each other in the ‘fault’ configuration, oriented along the long axis of the electrode (as in Carmichael et al., figure 1h^4^). Probe #3 was placed at the connector segment. Finally, the reference probe #4 was placed just beyond the ‘neck’ region of the phantom. Thermal stability was confirmed over 2 min prior to and following each 15‐min scan. Each scan was performed in both AP and RL orientations.

**Figure 3:**
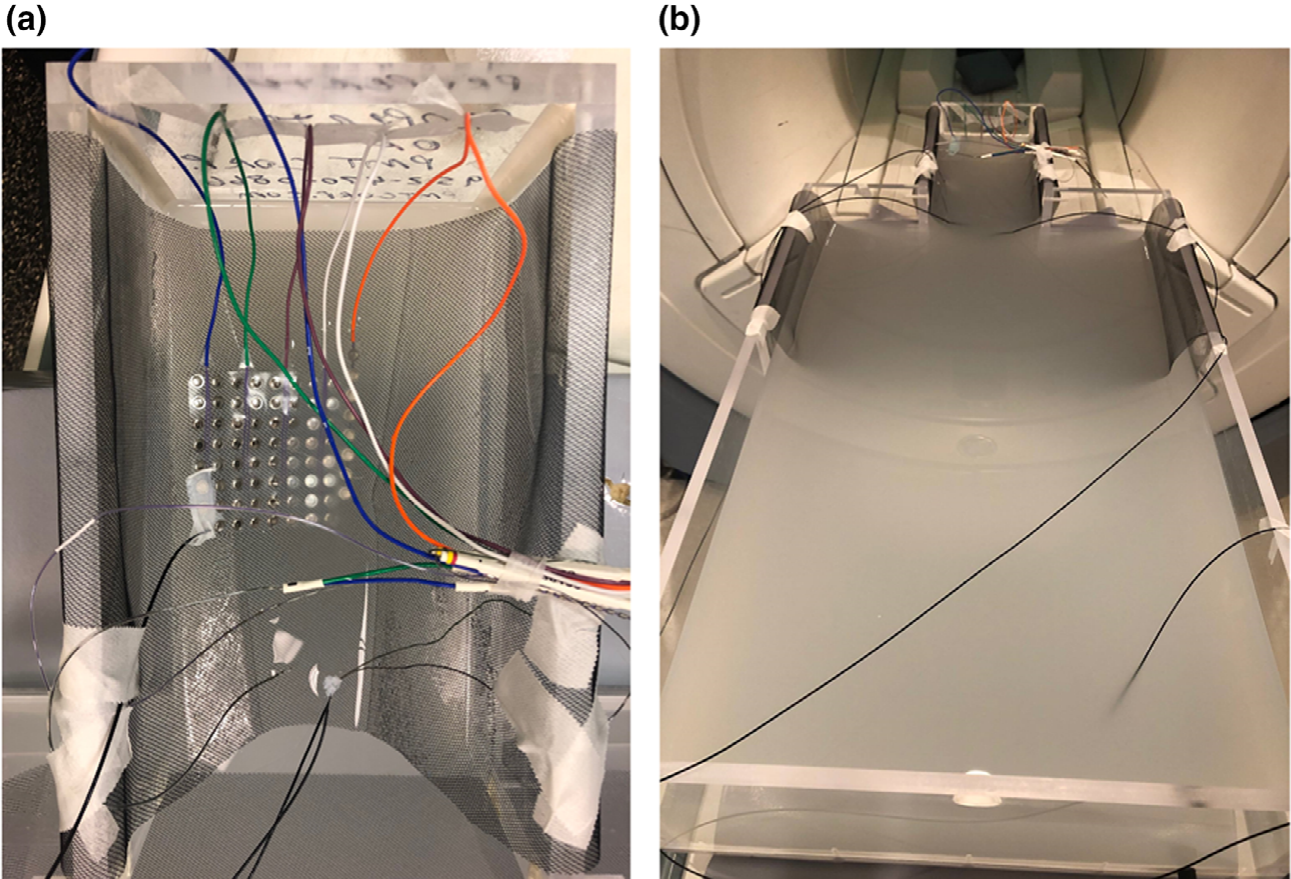
Experimental arrangement of test articles for simultaneous assessment of radiofrequency‐induced heating in simulation of an implant. (a) Picture showing arrangement of depth electrodes along the left–right axis and perpendicular to the sagittal plane, with the 16‐contact standard sEEG (ID# 2101‐16‐091) and 16‐contact RF ablation (ID# 2102‐16‐291) depth electrodes placed parallel to one another on the left side of the phantom ‘head’, and the 10‐contact depthalon (ID# 2102‐10‐084) and 8‐contact macro‐/16‐contact microwire (ID# 2104‐16‐16‐001) depth electrodes placed parallel to one another on the right; the subdural grid and strip electrodes were placed flat onto the mesh along the cranio‐caudal axis, with the tails oriented cranially in a configuration that simulated implants recording from the cortical surface. (b) Locations of the four MRI‐compatible fluoroptic thermometer probes. Probe #4 was placed just beyond the neck region of the phantom and was used as the reference; probe #1 was placed at the most distal contact (contact #1) of the 16‐contact standard eEEG depth electrode (2102‐16‐091); probe #2 was positioned on the most distal contact (contact #1) of the 16‐contact RF ablation depth electrode (ID# 2102‐16‐291); probe #3 was positioned on the corner contact (contact #48) of the 64‐contact grid electrode (ID# 2110‐64‐042).

Once all items had been tested individually, they were all placed into the phantom simultaneously to simulate an implantation, as described in Carmichael et al.^4^ and depicted in Figure 3a. Again, measurements were performed with tails in both ‘open’ and ‘fault’ configurations; tails were positioned outside of and parallel to the walls of the phantom in each case. Probe #1 was placed at the most distal contact (contact #1) of the 16‐contact standard sEEG depth electrode, while probe #2 was positioned on the most distal contact (contact #1) of the 16‐contact RF ablation depth electrode; these electrodes were placed in parallel on the left. Probe #3 was positioned on the corner contact (contact #48) of the 64‐contact grid electrode (Fig. 3b), and probe #4 was used as the reference. Initially, all contacts were separated during these tests; however, we repeated the ‘fault’ condition with the most distal contacts of the two left‐sided depth electrodes touching one another. Each scan was performed in both AP and RL orientations.

### Artefact

All articles were evaluated for MRI image artefacts in accordance with ASTM F2119‐07 – ‘Standard Test Method for Evaluation of MR Image Artifacts from Passive Implants’.^9^ Briefly, each article was suspended on a nylon netting within a plastic container filled with a 1.5 g/L solution of copper sulphate (CuSO_4_), ensuring at least 4 cm clearance from the walls of the container. An empty plastic pen was placed vertically into the solution as a reference (Fig. 2d). The container was moved to isocenter, and FSE images were generated in both AP and RL orientations.

## Results

### Displacement

All items passed both screening tests. The deflection angle was less than 45° in all cases (Table 1), indicating that the deflection force induced by the MR system's magnetic field was less than the force on the item due to gravity.

**Table 1:** Assessment of magnetically induced displacement force.

Test article	Trial	Displacement							
		Magnet	Phantom box	Length of string (cm)		Magnetic field at 1st movement (Gauss)		Deflection angle at 823 mm (degrees)	
				CM	Ends	CM	Ends	CM	Ends
16‐contact sEEG	1	Pass	Pass	8.0	21.5	1051	1685	10.0	9.0
ID#: 2102‐16‐091	2	Pass	Pass			930	1324	10.0	9.0
Lot #: 21519	3	Pass	Pass			988	1401	10.0	9.0
	Mean					990 ± 35	1470 ± 110	10.0 ± 0	9.0 ± 0
16‐contact sEEG (RF)	1	Pass	Pass	13.0	22.2	1231	1226	10.5	10.0
ID#: 2102‐16‐291	2	Pass	Pass			1139	1234	10.5	10.0
Lot #: 31519	3	Pass	Pass			1134	1241	10.5	10.0
	Mean					1168 ± 32	1234 ± 4	10.5 ± 0	10.0 ± 0
10‐contact depthalon	1	Pass	Pass	9.4	18.0	6280	9327	1.0	0.5
ID#: 2102‐10‐084	2	Pass	Pass			6231	8517	1.0	0.5
Lot #: 31519	3	Pass	Pass			5641	9162	1.0	0.5
	Mean					6051 ± 205	9002 ± 247	1.0 ± 0	0.5 ± 0
16‐contact depthalon (macro/microwire)	1	Pass	Pass	8.0	18.0	2146	1835	9.0	10.0
ID#: 2104‐16‐16‐001	2	Pass	Pass			1986	1942	9.8	10.0
Lot #: 31519	3	Pass	Pass			2086	1684	9.5	9.5
	Mean					2073 ± 47	1820 ± 75	9.4 ± 0.2	9.8 ± 0.2
64‐contact grid	1	Pass	Pass	8.7	11.0	19,982	20,052	0.5	0.3
ID#: 2110‐64‐042	2	Pass	Pass			20,072	20,050	0.5	0.0
Lot #: 31519	3	Pass	Pass			20,023	20,072	0.5	0.3
	Mean					20,026 ± 26	20,058 ± 7	0.5 ± 0	0.2 ± 0.1
8‐contact strip	1	Pass	Pass	9.5	12.8	19,849	19,867	0.0	0.0
ID#: 2110‐08‐031	2	Pass	Pass			19,934	19,736	0.0	0.0
Lot #: 111015	3	Pass	Pass			19,945	19,964	0.0	0.0
	Mean					19,909 ± 30	19,856 ± 66	0.0 ± 0	0.0 ± 0
sEEG anchor bolt	1	Pass	Pass	26.0		6686		1.5	
ID#: 2103‐29‐40	2	Pass	Pass			5682		1.3	
Lot #: 102618	3	Pass	Pass			6211		1.5	
	Mean					6193 ± 290		1.4 ± 0.1	
Depthalon anchor bolt	1	Pass	Pass	26.0		6840		2.0	
ID#: 2103‐27‐40	2	Pass	Pass			7205		2.0	
Lot #: 22418	3	Pass	Pass			7180		2.0	
	Mean					7075 ± 118		2.0 ± 0	
Depthalon cap	1	Pass	Pass	30.0		10,158		1.0	
	2	Pass	Pass			10,118		1.3	
	3	Pass	Pass			10,134		1.3	
	Mean					10,137 ± 12		1.2 ± 0.1	
Depthalon stylet	1	Fail							

### Torque

No rotation was seen for any item or portion thereof apart from the Depthalon cap, which was graded +2 (Table 2a). Coefficients of friction are reported in Table 2a. The maximum magnetically induced torque on the contact and connector segments was obtained using the lowest μ between the Perspex and Tyvek® surfaces; the maximal torque for the item as a whole was estimated using the highest μ between the contact and connector segments. The maximum magnetically induced torque was smaller than the worst‐case torque induced by gravity on the item in all cases, apart from the 8‐contact strip considered as a whole and for its contact segment alone (Table 2b). In both cases, the maximum torque was 11% greater.

**Table 2:** Assessment of magnetically induced torque.

(a)									
Test article	Trial	Torque				Coefficient of friction			
		Perspex		Tyvek		Perspex		Tyvek	
		Contacts	Connector	Contacts	Connector	Contacts	Connector	Contacts	Connector
16‐contact sEEG	1	Pass	Pass	Pass	Pass	0.78	1.04	0.75	0.70
ID#: 2102‐16‐091	2	Pass	Pass	Pass	Pass	0.78	0.73	0.73	0.67
Lot #: 21519	3	Pass	Pass	Pass	Pass	0.81	0.73	0.75	0.75
	Mean					0.79	0.82	0.74	0.71
16‐contact sEEG (RF)	1	Pass	Pass	Pass	Pass	1.07	0.84	0.73	0.62
ID#: 2102‐16‐291	2	Pass	Pass	Pass	Pass	0.78	0.78	0.90	0.70
Lot #: 31519	3	Pass	Pass	Pass	Pass	0.75	0.93	0.97	0.73
	Mean					0.86	0.85	0.86	0.68
10‐contact depthalon	1	Pass	Pass	Pass	Pass	0.62	0.90	0.62	0.53
ID#: 2102‐10‐084	2	Pass	Pass	Pass	Pass	0.65	1.23	0.58	0.36
Lot #: 31519	3	Pass	Pass	Pass	Pass	0.62	1.04	0.49	0.49
	Mean					0.63	1.05	0.56	0.46
16‐contact depthalon (macro/microwire)	1	Pass	Pass	Pass	Pass	1.00	0.60	0.78	0.67
ID#: 2104‐16‐16‐001	2	Pass	Pass	Pass	Pass	0.78	0.62	0.73	0.70
Lot #: 31519	3	Pass	Pass	Pass	Pass	0.70	0.49	0.70	0.62
	Mean					0.82	0.57	0.74	0.67
64‐contact grid	1	Pass	Pass	Pass	Pass	0.93	0.67	0.87	0.73
ID#: 2110‐64‐042	2	Pass	Pass	Pass	Pass	0.70	0.67	0.90	0.67
Lot #: 31519	3	Pass	Pass	Pass	Pass	0.97	0.78	1.00	0.60
	Mean					0.86	0.71	0.92	0.67
8‐contact strip	1	Pass	Pass	Pass	Pass	1.07	0.78	1.28	0.90
ID#: 2110‐08‐031	2	Pass	Pass	Pass	Pass	1.07	0.84	1.28	0.87
Lot #: 111015	3	Pass	Pass	Pass	Pass	1.23	0.78	1.48	0.84
	Mean					1.12	0.80	1.34	0.87
sEEG anchor bolt	1	Pass		Pass		0.31		0.31	
ID#: 2103‐29‐40	2	Pass		Pass		0.34		0.34	
Lot #: 102618	3	Pass		Pass		0.32		0.27	
	Mean					0.32		0.31	
Depthalon anchor bolt	1	Pass		Pass		0.49		0.21	
ID#: 2103‐27‐40	2	Pass		Pass		0.42		0.25	
Lot #: 22418	3	Pass		Pass		0.45		0.21	
	Mean					0.45		0.22	
Depthalon cap	1	+2		+2		0.38		0.38	
	2	+2		+2		0.42		0.40	
	3	+2		+2		0.45		0.40	
	Mean					0.42		0.40	
Depthalon stylet	NT								

(b)												
Test article	Length (mm)			Weight (g)			Max magnetically induced torque (N m)			Worst‐case torque due to gravity (N m)		
	Total	Contacts	Connector	Total	Contacts	Connector	Total	Contacts	Connector	Total	Contacts	Connector
16‐contact sEEG	392	57	80	1.67	0.14	1.39	4.78E‐03	5.82E‐05	7.73E‐04	6.42E‐03	7.82E‐05	1.09E‐03
16‐contact sEEG (RF)	336	57	81	1.73	0.15	1.44	4.89E‐03	7.20E‐05	7.81E‐04	5.70E‐03	8.38E‐05	1.14E‐03
10‐contact depthalon	461	100	46	1.55	1.27	0.2	3.94E‐03	6.99E‐04	4.14E‐05	7.00E‐03	1.24E‐03	9.02E‐05
16‐contact depthalon (macro/microwire)	427	58.25	80	3.6	0.38	3.04	1.11E‐02	1.60E‐04	1.59E‐03	1.51E‐02	2.17E‐04	2.38E‐03
64‐contact grid	560	74.5	65	15.1	9.57	4.06	7.12E‐02	6.00E‐03	1.83E‐03	8.29E‐02	6.99E‐03	2.59E‐03
8‐contact strip	522	74.5	32	2.19	2.01	0.16	1.26E‐02	1.65E‐03	4.02E‐05	1.12E‐02	1.47E‐03	5.02E‐05
sEEG anchor bolt	40			0.61			7.31E‐05			2.39E‐04		
Depthalon anchor bolt	40			2.03			1.79E‐04			7.96E‐04		
Depthalon cap	5			0.47			9.15E‐06			2.30E‐05		
Depthalon stylet												

*Note*: Pass, no torque; +1, slight change in orientation, but no alignment to magnetic field; +2, gradual alignment to magnetic field; +3, rapid and forceful alignment to magnetic field; +4, very rapid and very forceful alignment to magnetic field.

### Radiofrequency‐induced heating

The maximum change in temperature from baseline (Δ*T*_max_) for each component of each item and in the implantation simulation was invariably less than 2° across all conditions (Table 3). The greatest Δ*T*_max_ (1.7°C) was observed at the connector of the 8‐contact strip in the ‘open’ tail configuration, in the AP phase. Plots of change in temperature from baseline over time at each component of each item are shown in Figures [Link], [Link], and at each element of the implantation configuration in Figure S10, in both ‘open’ and ‘fault’ configurations and in both AP and RL phases.

**Table 3:** Assessment of radiofrequency‐induced heating.

Test article	Thermometer probe location	Δ*T*_max_ (°C)			
		Open		Fault	
		AP	RL	AP	RL
16‐contact sEEG	Contact 1	0.7	0.8	0.6	0.7
ID#: 2102‐16‐091	Tail	0.2	0.5	1.2	1.3
Lot #: 21519	Connector	1.4	1.6	1.0	1.0
	Reference	0.1	0.1	0.1	0.2
16‐contact sEEG (RF)	Contact 1	0.2	0.3	0.5	0.6
ID#: 2102‐16‐291	Tail	0.3	0.1	0.4	0.5
Lot #: 31519	Connector	0.3	0.2	0.5	0.4
	Reference	0.1	0.1	0.2	0.1
10‐contact depthalon	Contact 1	0.6	0.5	0.2	0.2
2102‐10‐084	Tail	1.2	0.9	0.7	0.5
Lot #: 31519	Connector	1.4	1.2	0.6	0.6
	Reference	0.1	0.2	0.1	0.1
16‐contact depthalon (macro/microwire)	Contact 1	0.2	0.4	0.2	0.2
2104‐16‐16‐001	Tail	1.5	0.9	0.4	0.4
Lot #: 31519	Connector	0.5	0.3	0.4	0.3
	Reference	0.2	0.0	0.1	0.1
64‐contact grid	Contact 1	0.1	0.1	0.2	0.1
2110‐64‐042	Tail	0.4	0.2	1.1	1.1
Lot #: 31519	Connector	0.3	0.2	0.0	0.1
	Reference	0.2	0.1	0.1	0.1
8‐contact strip	Contact 1	1.2	1.1	0.6	0.7
2110‐08‐031	Tail	1.3	1.0	1.0	0.9
Lot #: 111015	Connector	1.7	1.5	1.6	1.5
	Reference	0.0	0.1	0.1	0.1
Implantation	Contact 1 (2101‐16‐091)	0.1	0.1	0.1	0.2
(separated)	Contact 1 (2102‐16‐291)	0.3	0.2	0.3	0.4
	Contact 48 (2110‐64‐042)	0.2	0.2	0.7	0.8
	Reference	0.1	0.1	0.0	0.0
Implantation	Contact 1 (2101‐16‐091)			0.1	0.1
(direct contact)	Contact 1 (2102‐16‐291)			0.6	0.7
	Contact 48 (2110‐64‐042)			0.6	0.6
	Reference			0.3	0.1

### Artefact

**Figure 4:**
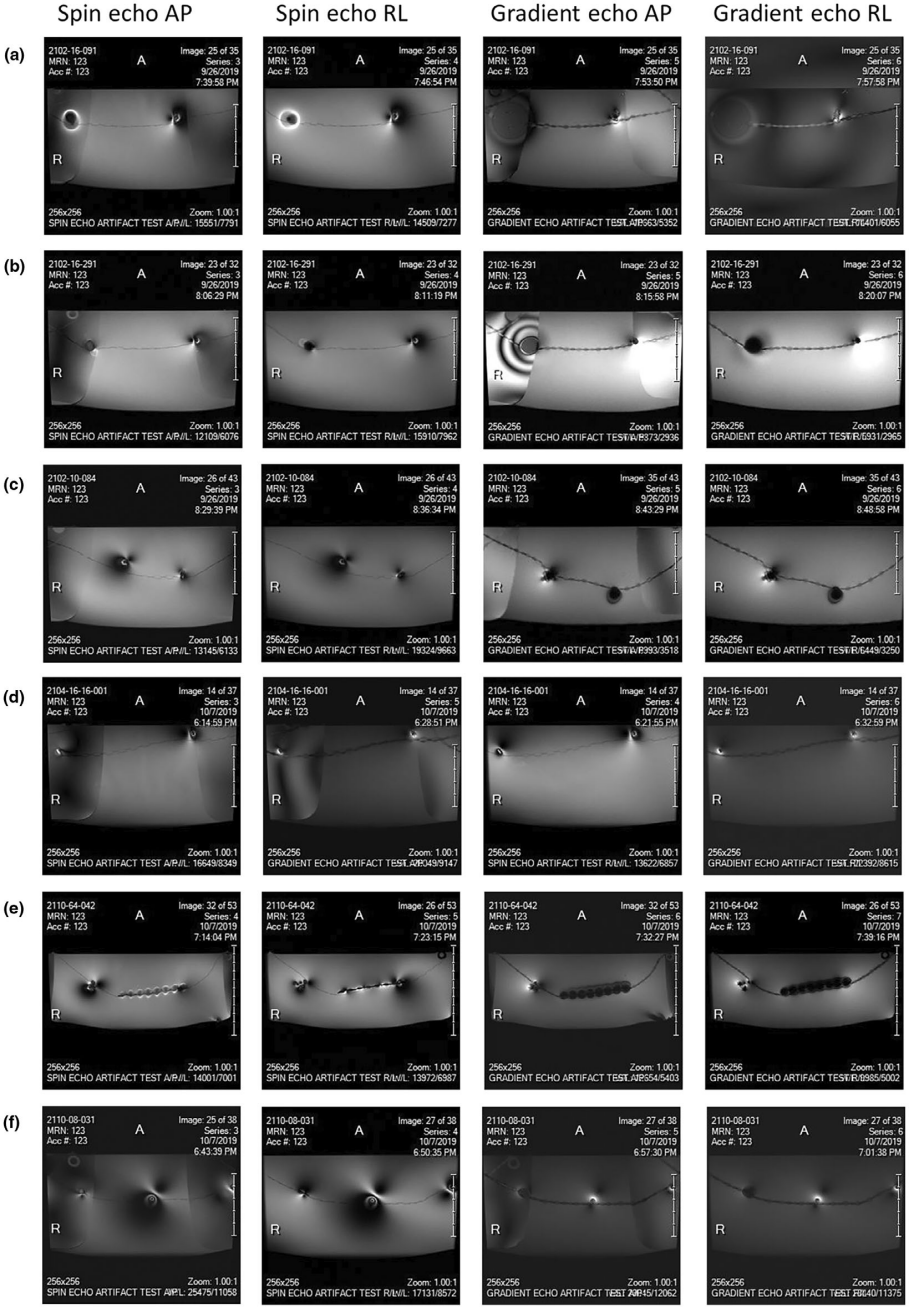
Images of the expected distortion and signal loss artefacts produced in MR imaging of test articles. (a) 16‐contact standard depth electrode ID# 2101‐16‐091, (b) 16‐contact RF ablation depth electrode ID# 2102‐16‐291, (c) 10‐contact depthalon depth electrode ID# 2102‐10‐084), (d) 8‐contact depth + 16‐contact microwire electrode ID# 2104‐16‐16‐001, (e) 64‐contact Contac grid ID# 2110‐64‐042, and (f) 8‐contact Contac strip ID# 2110‐08‐031.

Images of the expected distortion and signal loss artefacts produced in MR imaging of each of the items are shown in Figure 4.

## Discussion

For all items, the magnetically induced displacement and torque forces were less than the force due to gravity, suggesting that the risk of electrode movement imposed by their presence within a static magnetic field is no greater than that imposed by the earth's gravitational field. The exceptions were the 8‐contact strip – for which it was 11% greater – and the depthalon cap. Torque 11% greater than that induced by gravity alone is similar to the torque applied on the head in normal daily activity, including sitting down, coughing, and shoulder‐checking.^10^ Meanwhile, the depthalon cap is meant to be threaded over an sEEG wire and screwed onto the depthalon anchor bolt and is thus unable to rotate freely. These data suggest that the user confirms that each cap is firmly affixed to the bolt prior to placing the device into an MRI environment.

When each item was tested individually, the greatest Δ*T*_max_ (1.7°C) was observed at the connector of the 8‐contact strip in the ‘open’ tail configuration, in the AP phase. However, this portion of the device is not implanted intracranially. The greatest Δ*T*_max_ for any intracranial component was 1.2°C, on the same 8‐contact strip in the same condition. According to thermal threshold guidelines for the safe use of MRI during standard examinations (i.e. those posing ‘no or minimal risk’ of adverse events),^11^ it is recommended that the maximum local temperature of any tissue be limited to 39°C in all patients, including those with fever, impaired thermoregulation, and implants. Accordingly, RF‐induced heating should be limited to no more than 2°C. All components of all test articles examined in this report met this threshold individually and in the ‘implantation simulation’, including the ‘fault’ configurations.

Our measurements of RF‐induced heating broadly follow the principles of ASTM F2182‐2011^5^; a standard for testing MRI‐induced temperature increases near passive elongated implants. This represents a worst‐case scenario as in contrast to human tissue where blood flow redistributes heat in the body; there is no vascularization in the test phantom. With this in mind, although we believe our results provide a reasonable estimate of the worst‐case temperature change induced by RF heating, the field distributions within our phantom may not accurately represent those found within a human body.^4^, ^12^, ^13^, ^14^ Moreover, although temperature changes are highly reproducible for a given lead in a given location, differences in electrode position within the phantom, as well as the location of the phantom,^4^ mean that we cannot exclude the possibility that greater heating than reported herein occurred at electrode or phantom positions we were unable to monitor. However, we addressed this by testing various electrode configurations under both open and fault configurations and in both AP and RL phases to increase the likelihood of testing the worst‐case configuration. In addition, we performed our assessment in an ‘implantation simulation’ with multiple implanted leads, to test for interaction between different electrodes. The location and orientation of the temperature probes may also be a source of variability. The locations of the probes were determined based on pilot observations which showed the temperature rise to be maximal at these locations. Moreover, we positioned our temperature probes in a transverse orientation relative to the electrode contacts, which gave the highest peak temperature change in a prior study of pacemaker electrodes.^4^ Nonetheless, variations in implant location and/or configuration between individual patients, or deviations from a standard supine patient position relative to the scanner bore, may lead to changes in temperature which are not reflected in the current study. Finally, the electrodes evaluated in this report are intended for use as passive implants. Thus, they are not to be connected to a power source during MRI scanning and have not been evaluated under this condition.

The above studies were all performed on a 3.0T GE Signa HDxT MRI system. Notably, prior studies revealed that two different‐generation MRI systems from the same manufacturer, using similar coils and RF‐deposited energy, were associated with significantly different temperature changes, suggesting that RF‐induced heating may be inconsistent across MRI scanners, software, and deep brain stimulation (DBS) device configurations.^15^, ^16^ The implication is that calculated whole‐body averaged SAR may not be a reliable metric for RF power irradiation across different MR systems. Therefore, our findings may not be generalizable to other MRI scanners. We chose a 15‐min scan time for our test sequences as we felt that this would exceed most uninterrupted sequences which might be performed in sEEG electrode localization. While the temperature rise seen in our experiments was broadly stable after an initial increase typically seen within the first minute of scanning, sequences of substantially longer duration should be avoided.

## Conclusions

As with previous studies on iEEG electrodes from a different manufacturer,^4^, ^12^, ^17^, ^18^, ^19^, ^20^, ^21^ this study suggests that MRIs can be performed safely at 3.0T to localise iEEG electrodes under certain conditions. The current study was performed on a 3.0T GE Signa HDxT MRI system, and these results may not be generalizable to other systems. Moreover, as prior studies demonstrate that head transmit–receive coils result in significantly less localised heating compared to body coils,^4^, ^12^, ^22^, ^23^, ^24^, ^25^, ^26^ we recommend the use of a head transmit–receive RF coil in patients undergoing MR imaging with iEEG electrodes. As for the electrodes themselves, all components including the anchors, wires, and caps should be secured in place prior to scanning. Furthermore, despite there being no consistent difference between the ‘open’ and ‘fault’ conditions in our study, we recommend that electrode tails be separated in order to avoid electrically conductive loops, and that all devices be disconnected, in accordance with the manufacturer's recommendations. Prior publications further recommend placing all leads and cables along the scanner's central axis, extending posteriorly from the bore.^12^, ^17^ Finally, our measurements are representative of our local surgical and imaging practices, which we feel are consistent with the most common practices in centres performing invasive intracranial monitoring. However, any arrangements which substantially differ from those depicted in the current study and from typical practice may require further specific safety investigation.

Of note, the manufacturer of the electrodes used in this work (PMT Corporation) does not have, or claim, US Food and Drug Administration (FDA)‐approved MR‐Conditional status for these products, or European certification for safe use with MRI.

## Conflict of Interest

YBB is a paid consultant for PMT Corporation. This study was funded by PMT Corporation.

## Supporting information


**Figure S1.** Schematic of 16‐contact standard depth electrode ID# 2101‐16‐091.

**Figure S2.** Schematic of 16‐contact RF ablation depth electrode ID# 2102‐16‐291.

**Figure S3.** Schematic of 10‐contact depthalon depth electrode ID# 2102‐10‐084.

**Figure S4.** Schematic of 8‐contact depth + 16‐contact microwire electrode ID# 2104‐16‐16‐001.

**Figure S5.** Schematic of 64‐contact Contac grid ID# 2110‐64‐042.

**Figure S6.** Schematic of 8‐contact Contac strip ID# 2110‐08‐031.

**Figure S7.** Plots of change in temperature from baseline over time at each component of each test article are shown in both open and fault configurations and obtained in both the AP and RL phases. 16 contact standard depth electrode ID# 2101‐16‐091 in the (a) open/AP, (b) open/RL, (c) fault/AP, and (d) fault/RL configurations; and 16 contact RF ablation depth electrode ID# 2102‐16‐291 in the (e) open/AP, (f) open/RL, (g) fault/AP, and (h) fault/RL configurations.

**Figure S8.** Plots of change in temperature from baseline over time at each component of each test article are shown in both open and fault configurations and obtained in both the AP and RL phases. 10 contact depthalon depth electrode ID# 2102‐10‐084 in the (a) open/AP, (b) open/RL, (c) fault/AP, and (d) fault/RL configurations; and 8 contact depth + 16 contact microwire electrode ID# 2104‐16‐16‐001 in the (e) open/AP, (f) open/RL, (g) fault/AP, and (h) fault/RL configurations.

**Figure S9.** Plots of change in temperature from baseline over time at each component of each test article are shown in both open and fault configurations and obtained in both the AP and RL phases. 64 contact Contac grid ID# 2110‐64‐042 in the (a) open/AP, (b) open/RL, (c) fault/AP, and (d) fault/RL configurations; and 8 contact Contac strip ID# 2110‐08‐031 in the (e) open/AP, (f) open/RL, (g) fault/AP, and (h) fault/RL configurations.

**Figure S10.** Plots of change in temperature from baseline over time at each element of the implantation configuration.

## Data Availability

The data that support the findings of this study are available from the corresponding author upon reasonable request.
