# Supplementary figures and images for: Safety of intracranial electrodes in an MRI environment: a technical report
Source: J Med Radiat Sci. 2024 Mar 11;71(3):461–73. doi: 10.1002/jmrs.775 (PMC11569409; doi:10.1002/jmrs.775)

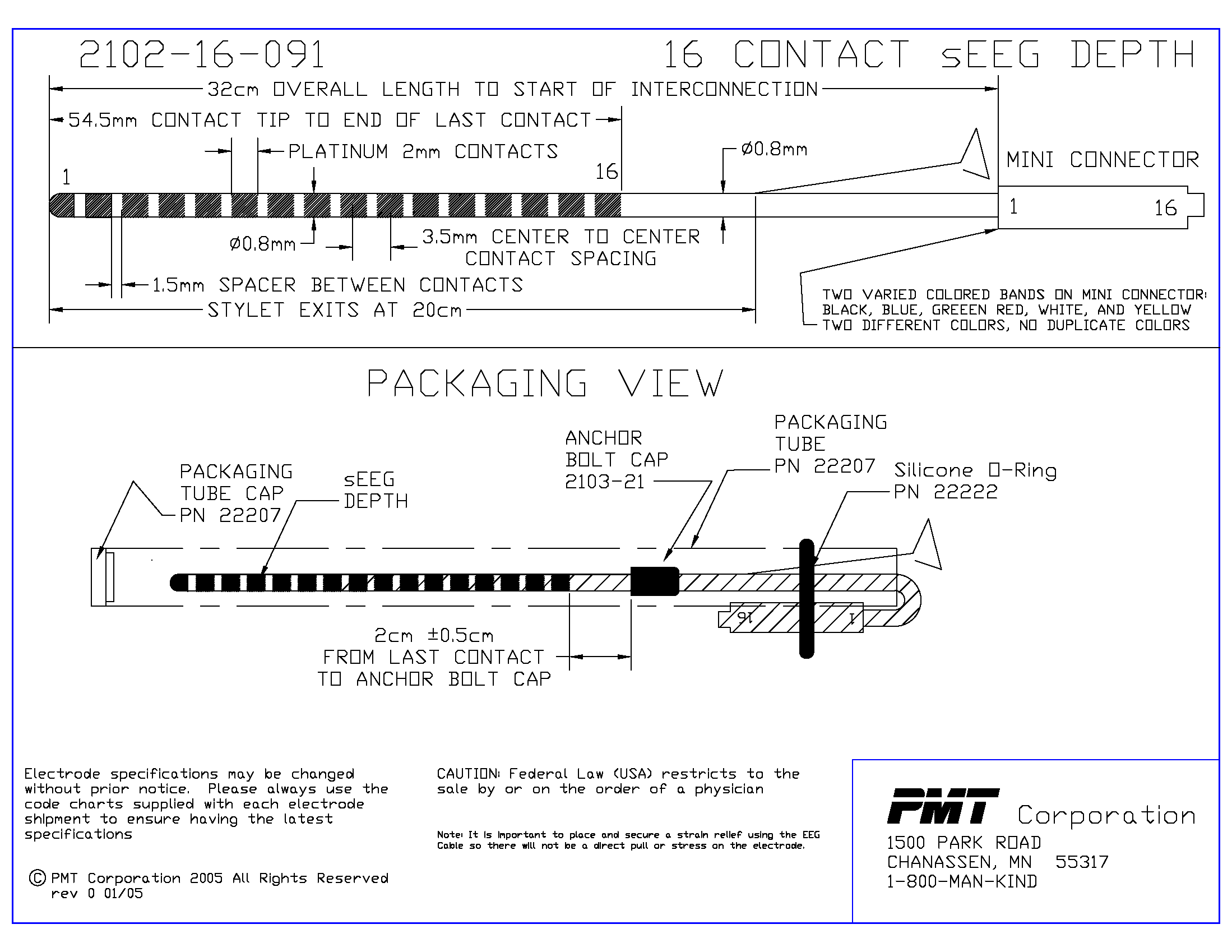

Supplement: Supplementary file 1 — Figure S1. Schematic of 16‐contact standard depth electrode ID# 2101‐16‐091. [file JMRS-71-461-s002.tiff]

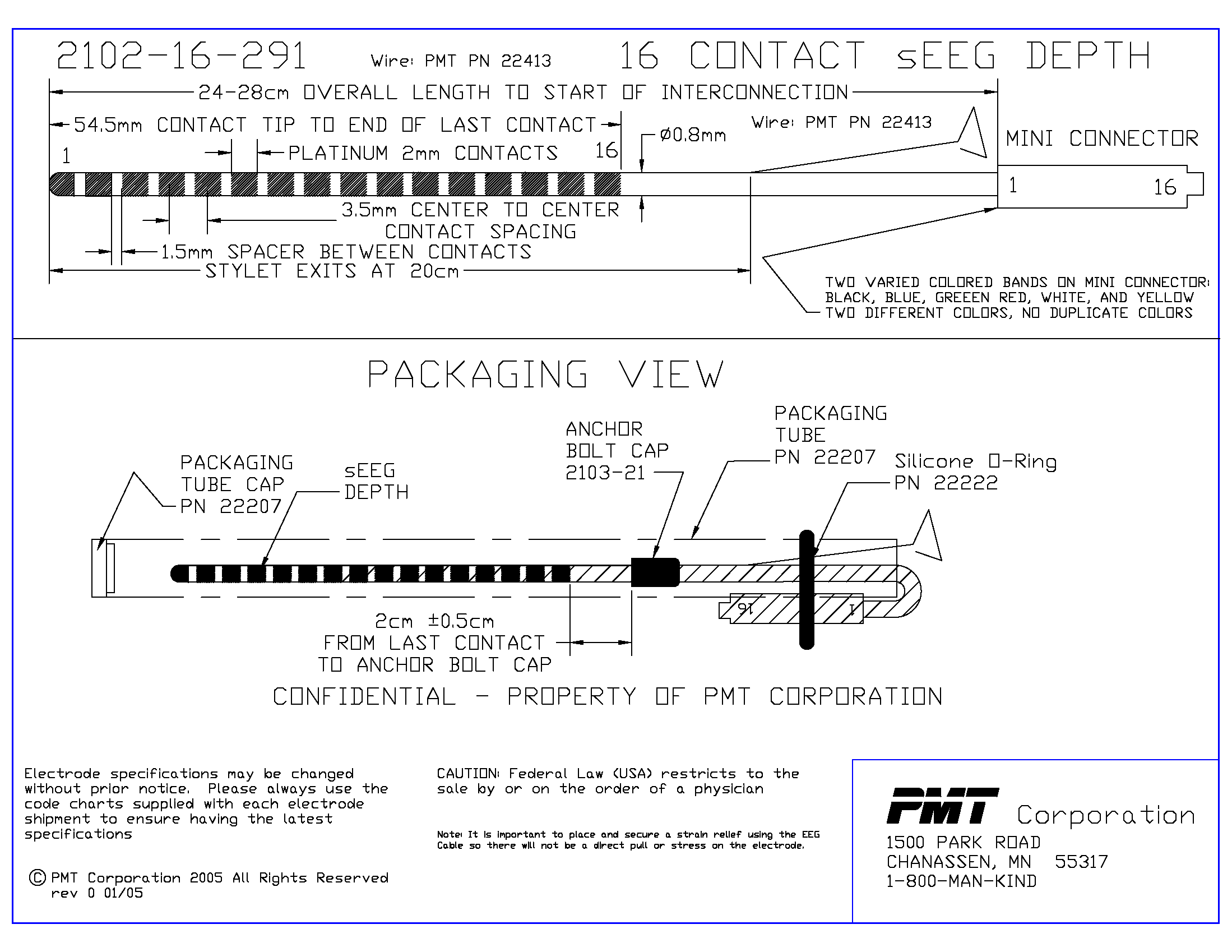

Supplement: Supplementary file 2 — Figure S2. Schematic of 16‐contact RF ablation depth electrode ID# 2102‐16‐291. [file JMRS-71-461-s007.tif]

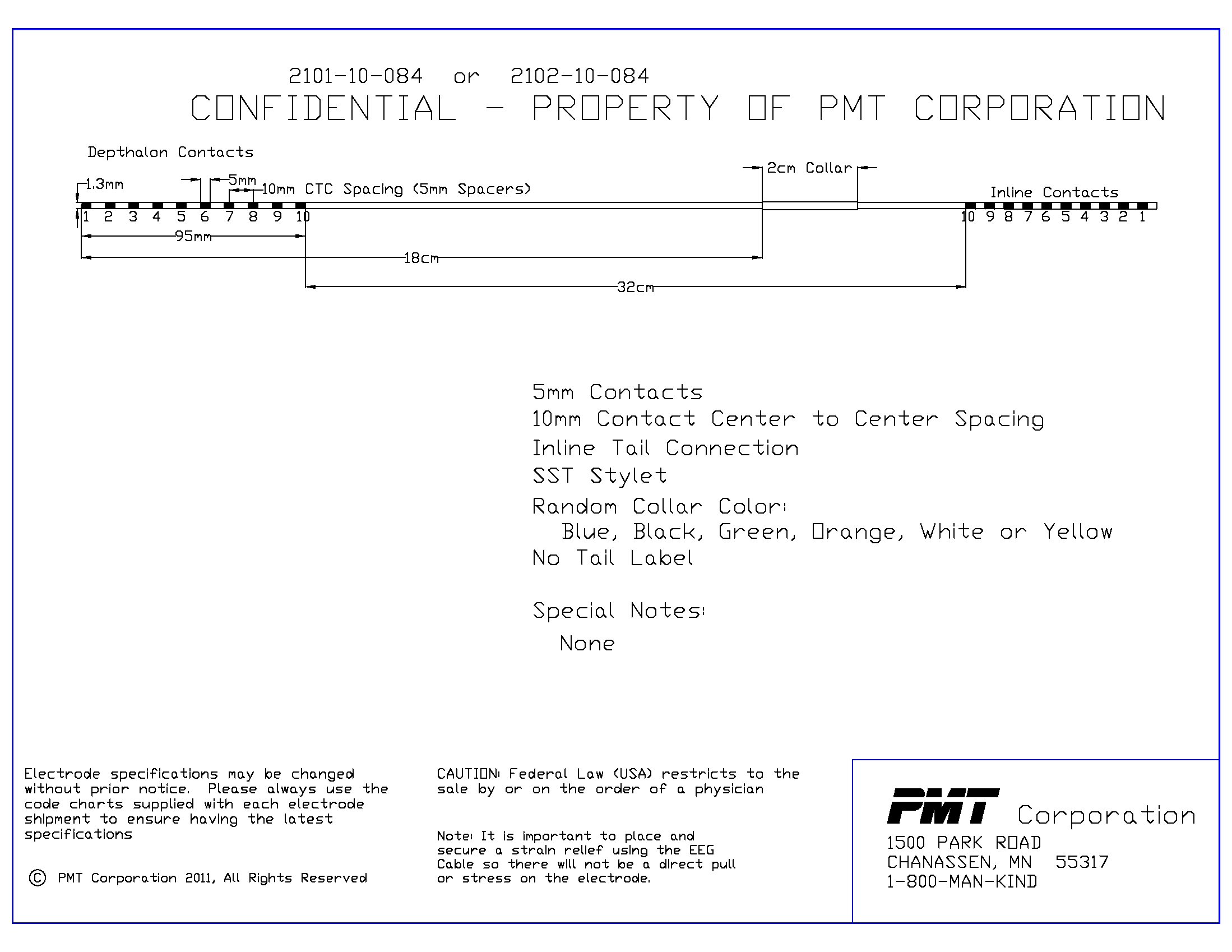

Supplement: Supplementary file 3 — Figure S3. Schematic of 10‐contact depthalon depth electrode ID# 2102‐10‐084. [file JMRS-71-461-s005.tiff]

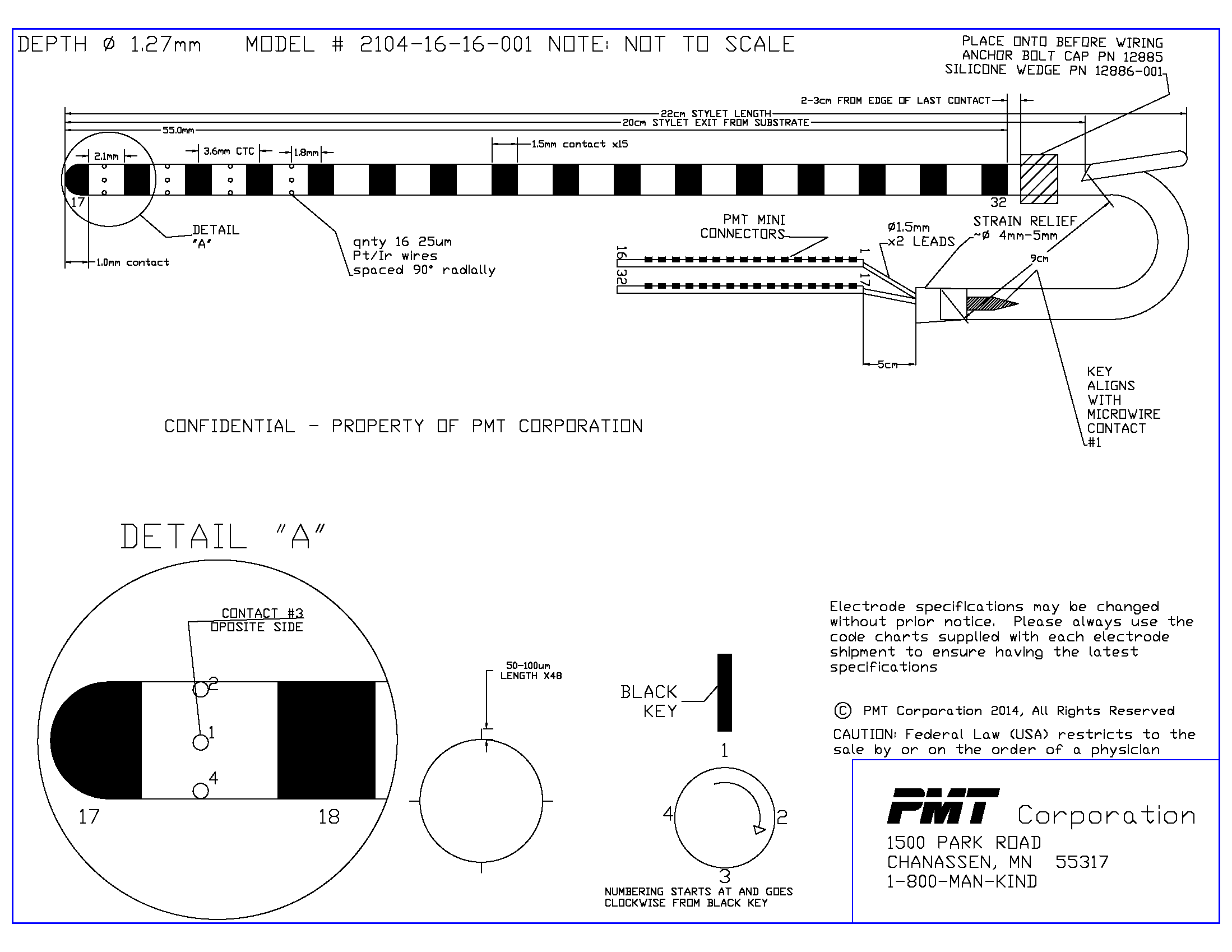

Supplement: Supplementary file 4 — Figure S4. Schematic of 8‐contact depth + 16‐contact microwire electrode ID# 2104‐16‐16‐001. [file JMRS-71-461-s004.tiff]

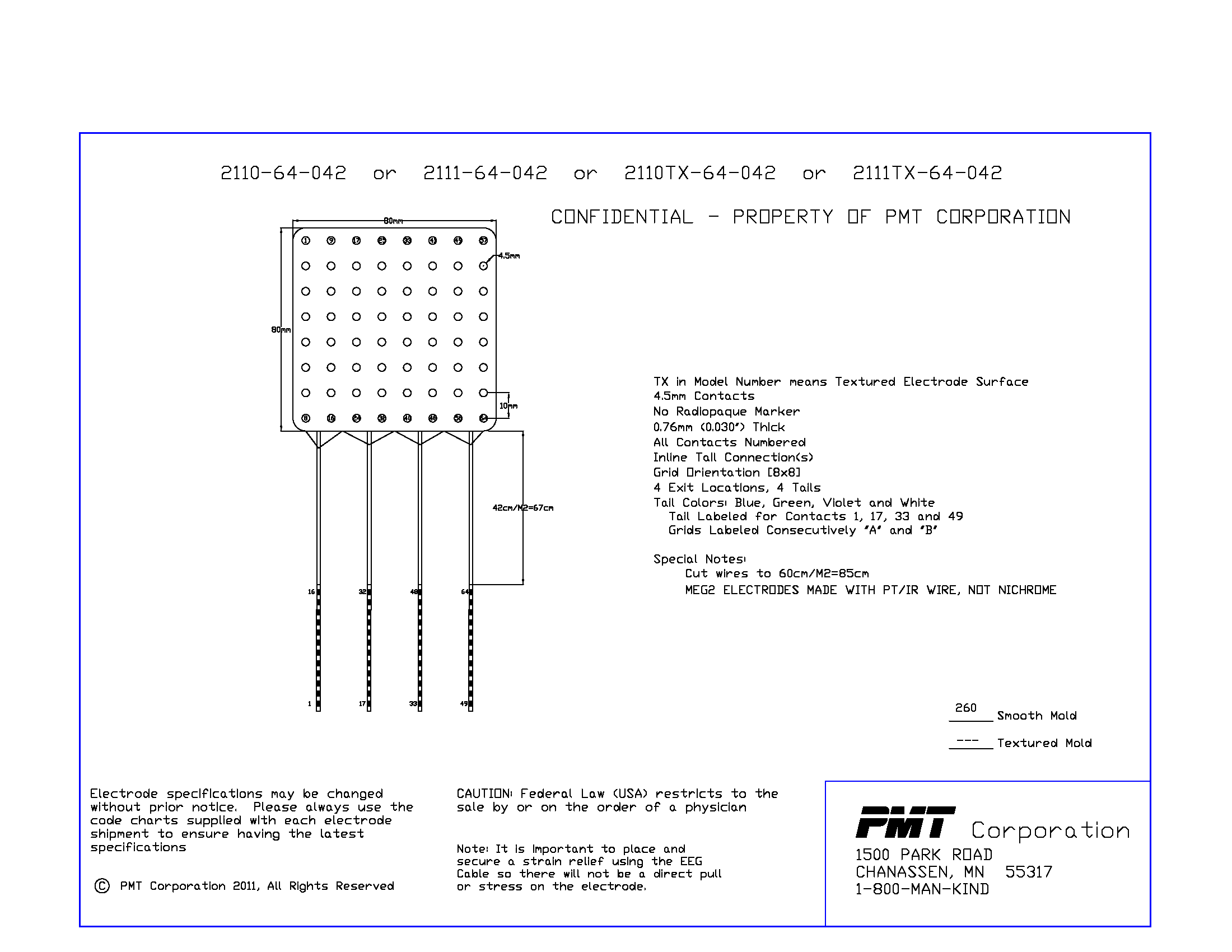

Supplement: Supplementary file 5 — Figure S5. Schematic of 64‐contact Contac grid ID# 2110‐64‐042. [file JMRS-71-461-s001.tiff]

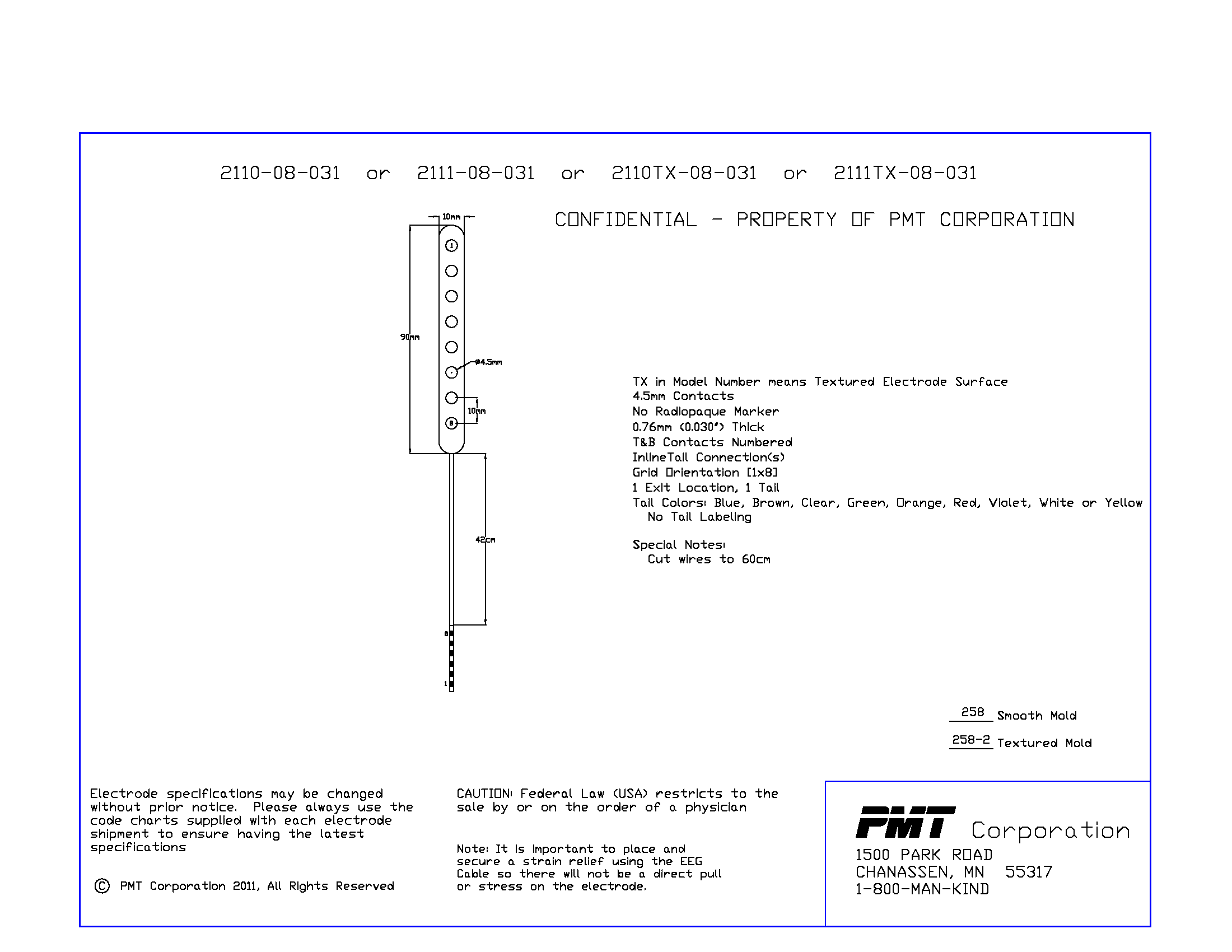

Supplement: Supplementary file 6 — Figure S6. Schematic of 8‐contact Contac strip ID# 2110‐08‐031. [file JMRS-71-461-s006.tiff]

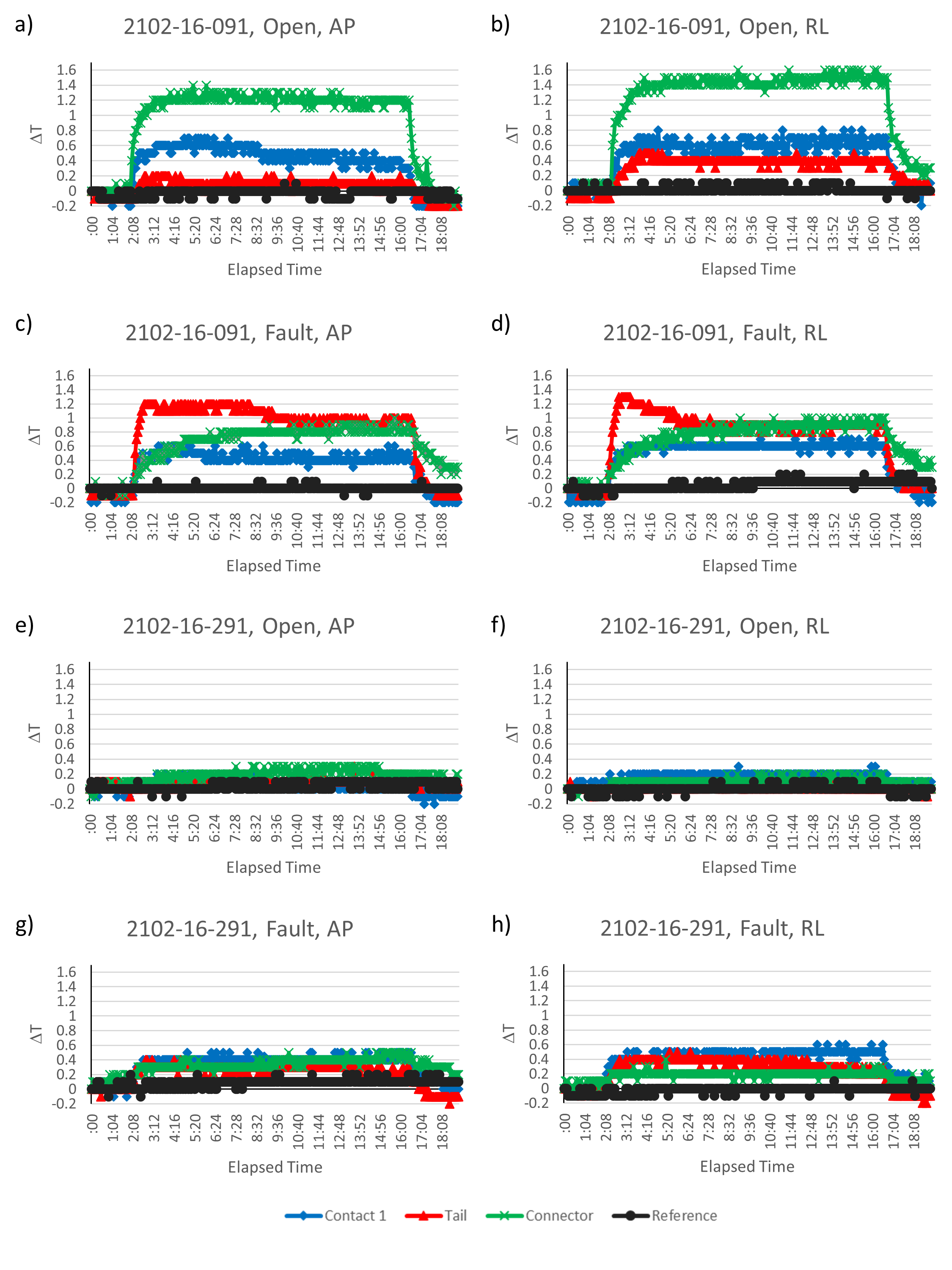

Supplement: Supplementary file 7 — Figure S7. Plots of change in temperature from baseline over time at each component of each test article are shown in both open and fault configurations and obtained in both the AP and RL phases. 16 contact standard depth electrode ID# 2101‐16‐091 in the (a) open/AP, (b) open/RL, (c) fault/AP, and (d) fault/RL configurations; and 16 contact RF ablation depth electrode ID# 2102‐16‐291 in the (e) open/AP, (f) open/RL, (g) fault/AP, and (h) fault/RL configurations. [file JMRS-71-461-s008.tif]

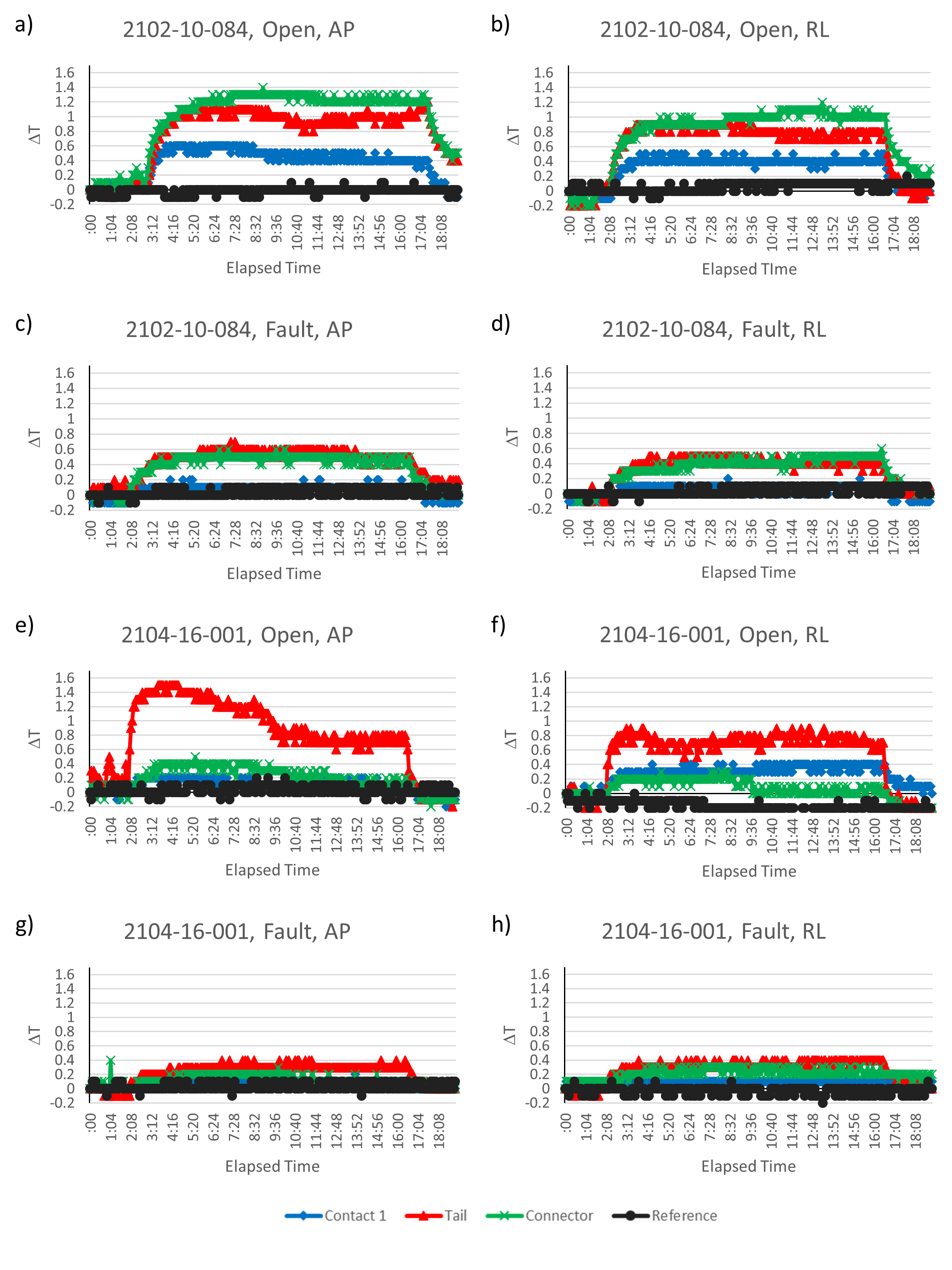

Supplement: Supplementary file 8 — Figure S8. Plots of change in temperature from baseline over time at each component of each test article are shown in both open and fault configurations and obtained in both the AP and RL phases. 10 contact depthalon depth electrode ID# 2102‐10‐084 in the (a) open/AP, (b) open/RL, (c) fault/AP, and (d) fault/RL configurations; and 8 contact depth + 16 contact microwire electrode ID# 2104‐16‐16‐001 in the (e) open/AP, (f) open/RL, (g) fault/AP, and (h) fault/RL configurations. [file JMRS-71-461-s003.tif]

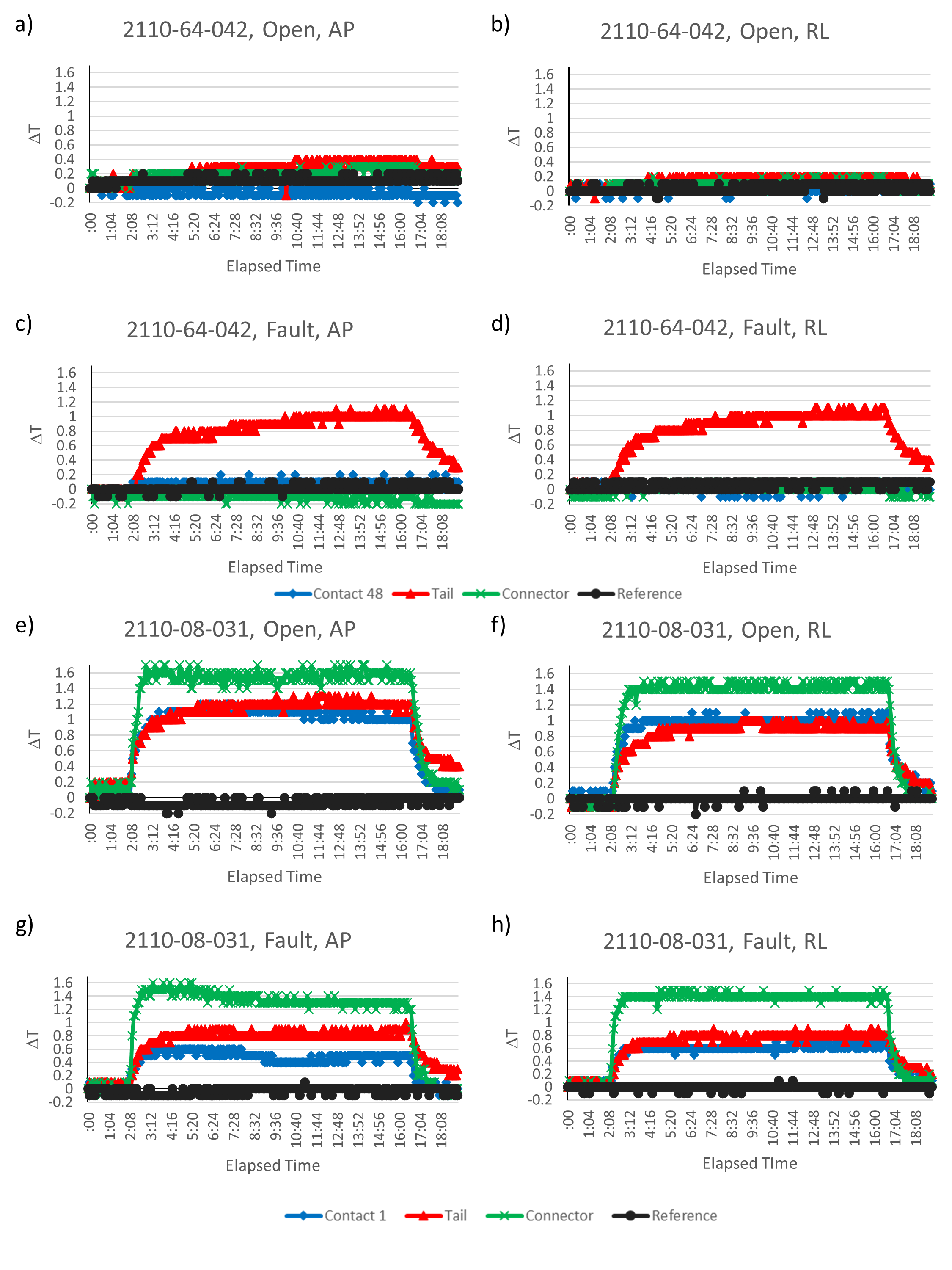

Supplement: Supplementary file 9 — Figure S9. Plots of change in temperature from baseline over time at each component of each test article are shown in both open and fault configurations and obtained in both the AP and RL phases. 64 contact Contac grid ID# 2110‐64‐042 in the (a) open/AP, (b) open/RL, (c) fault/AP, and (d) fault/RL configurations; and 8 contact Contac strip ID# 2110‐08‐031 in the (e) open/AP, (f) open/RL, (g) fault/AP, and (h) fault/RL configurations. [file JMRS-71-461-s010.tif]

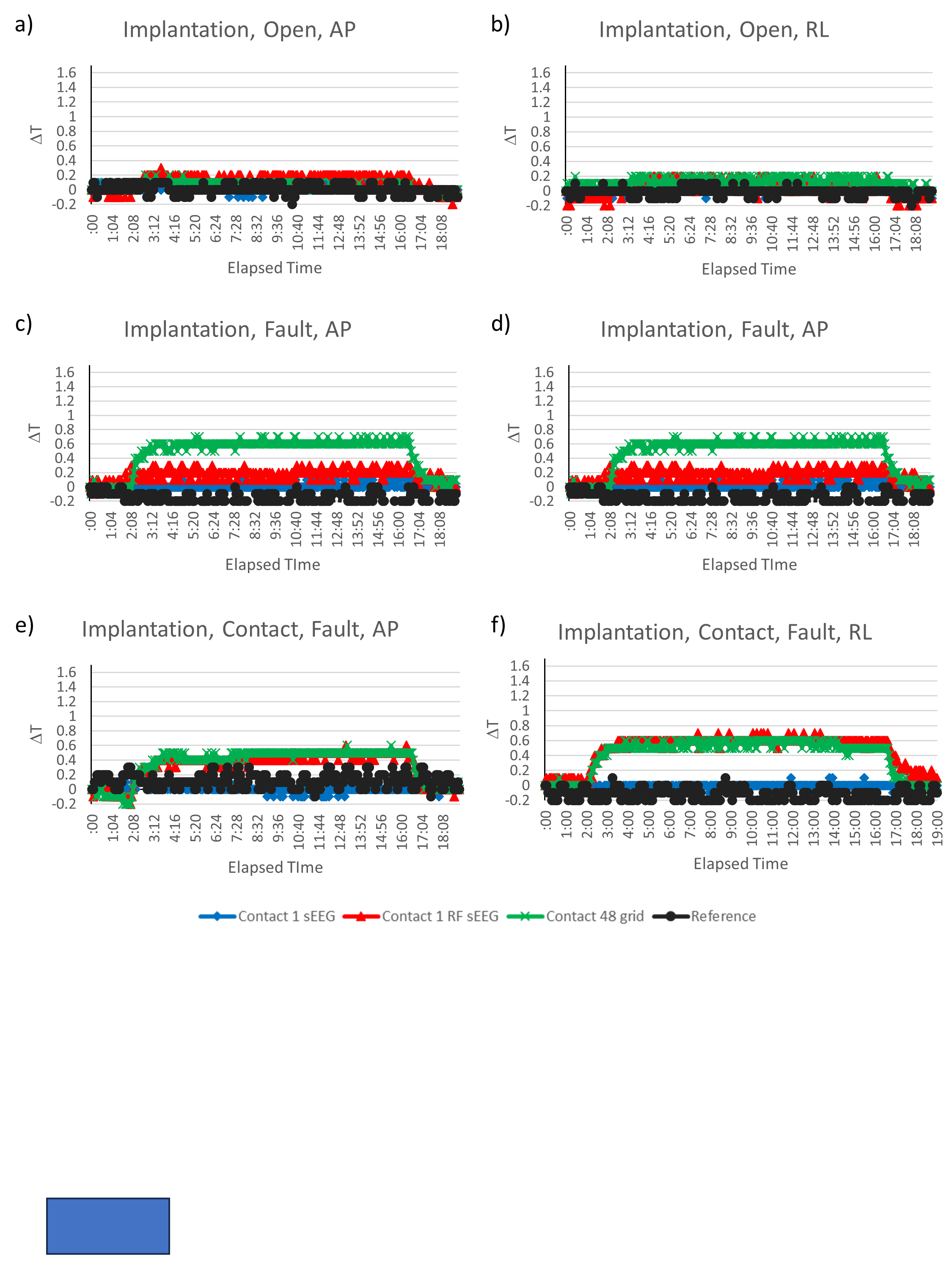

Supplement: Supplementary file 10 — Figure S10. Plots of change in temperature from baseline over time at each element of the implantation configuration. [file JMRS-71-461-s009.tiff]
